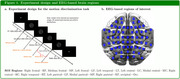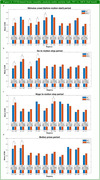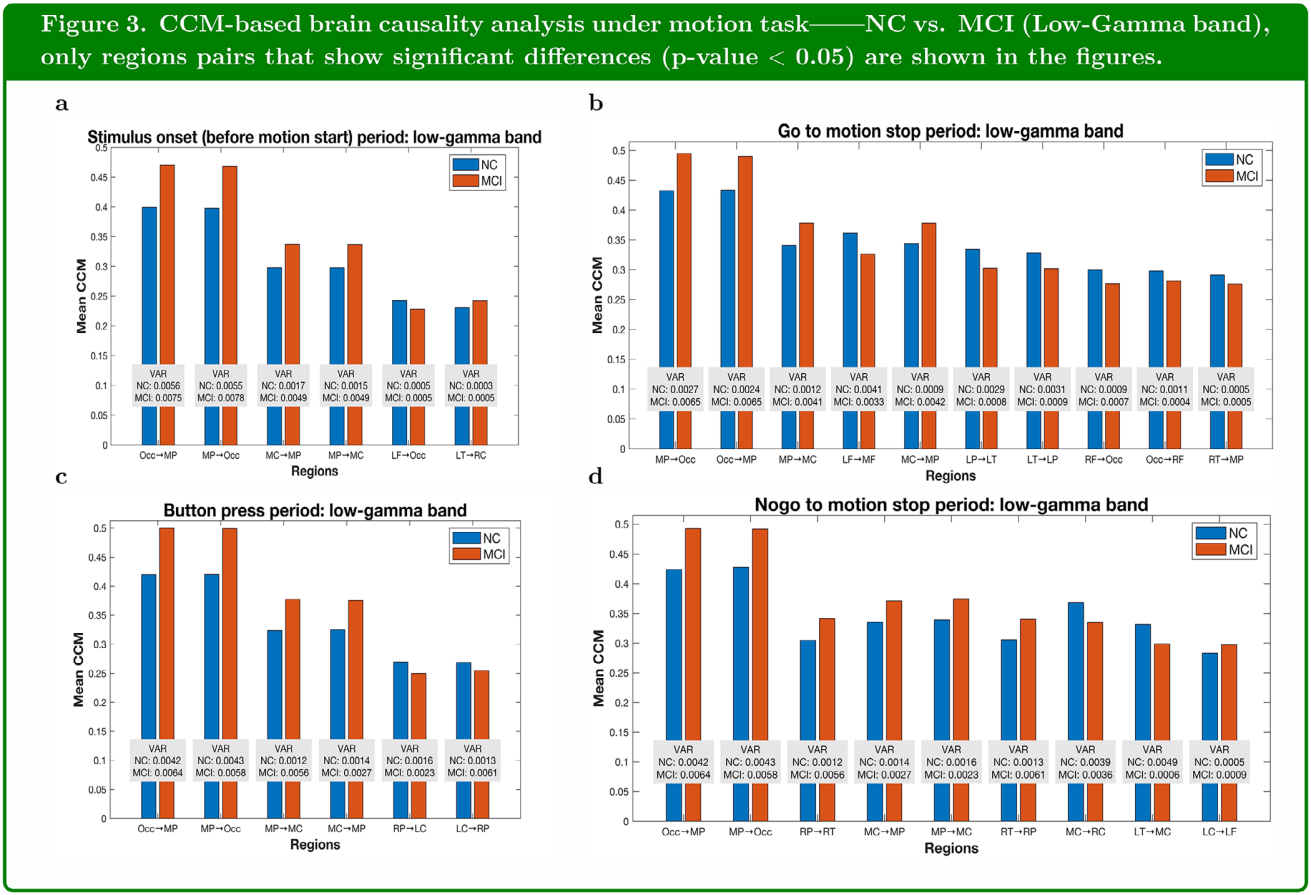# Altered Effective Connectivity Patterns in Low‐Gamma Band for MCI Patients in a Motion Detection Task

**DOI:** 10.1002/alz.088088

**Published:** 2025-01-09

**Authors:** Alina Brighty Renli, Boxin Sun, Ming Gu, Jinxian Deng, Voyko Kavcic, Tongtong Li, Bruno Giordani, Michigan Alzheimer's Disease Research Center

**Affiliations:** ^1^ Department of Neuroscience, Michigan State University, East Lansing, MI USA; ^2^ Michigan State University, East Lansing, MI USA; ^3^ Wayne State University, Detroit, MI USA; ^4^ International Institute of Applied Gerontology, Ljubljana Slovenia; ^5^ Michigan Alzheimer's Disease Research Center, Ann Arbor, MI USA; ^6^ University of Michigan Medical School, Ann Arbor, MI USA

## Abstract

**Background:**

Changes in effective connectivity, which represents the directed connectivity or information flow from one brain region to the other, have been proposed to underlie mild cognitive impairment (MCI) and Alzheimer’s disease (AD) pathology. The present study explores possible differences in brain effective connectivity between persons with normal cognition (NC) and patients with MCI.

**Method:**

Our research focuses on task‐based EEG (64‐channel) acquired at Wayne State University, where participants were asked to perform a motion direction discrimination task. The current dataset includes 56 consensus‐diagnosed, community‐dwelling African Americans with subjective cognitive complaints (ages 60‐90 years, 28 NC and 28 MCI) recruited through the Wayne State Institute of Gerontology and Michigan Alzheimer’s Disease Research Center.

We evaluated the effective connectivity at different time periods of the motion‐detection task across all the possible EEG region pairs using causalized convergent cross‐mapping (cCCM) of the current source density. For each task trial, the successive time periods being examined included: (I) stimulus onset to Go/No‐Go indication, (II) Go (or No‐Go) indication to motion‐stop, and (III) the button‐press period.

**Result:**

Our analysis indicated that MCI patients showed increased bidirectional effective connectivity in low‐gamma band (30‐48Hz) over MP (medial parietal)—Occ (occipital) and MP—MC (medial central) pairs in all the time periods during the motion task.

**Conclusion:**

Altered effective connectivity measures in low‐gamma band may reflect compensatory brain activity among older individuals with MCI as they struggle to achieve comparable behavioral results to NC during the motion direction discrimination task. While effective connectivity may be decreased for MCI across certain region pairs (see Figures), a significant increase in effective connectivity in some other region pairs may be an indicator associated with AD and MCI pathology.

**Funding**: NSF‐2032709/Li; NIH‐1R21AG046637‐01A1/Kavcic; NIH‐1R01AG054484‐01A1/Kavcic; NIH‐P30AG072931/Paulson and NIH‐P30AG024824/Yung.